# Triaged Out of Care: How Carceral Logics Complicate a ‘Course of Care’ in Solitary Confinement

**DOI:** 10.3390/healthcare10020289

**Published:** 2022-02-01

**Authors:** Melissa Barragan, Gabriela Gonzalez, Justin Donald Strong, Dallas Augustine, Kelsie Chesnut, Keramet Reiter, Natalie A. Pifer

**Affiliations:** 1 Department of Sociology, California Polytechnic University, Pomona, CA 93407, USA; 2 Department of Public Administration, California State University, Dominguez Hills, Carson, CA 90747, USA; ggonzalezmartinez@csudh.edu; 3 Department of Criminology, Law & Society, University of California, Irvine, CA 92697, USA; jdstrong@uci.edu (J.D.S.); reiterk@uci.edu (K.R.); 4 Benioff Homelessness and Housing Initiative, UCSF Center for Vulnerable Populations, University of California, San Francisco, CA 94110, USA; dallas.augustine@ucsf.edu; 5 Vera Institute of Justice, Brooklyn, NY 11232, USA; kchesnut@vera.org; 6 Department of Criminology and Criminal Justice, University of Rhode Island, Kingston, RI 02881, USA; npifer@uri.edu

**Keywords:** incarceration, prison, dual loyalty, triage, corrections

## Abstract

Incarceration, along with its most restrictive iteration, solitary confinement, is an increasingly common experience in America. More than two million Americans are currently incarcerated, and at least one-fifth of incarcerated people will experience solitary confinement. Understanding the barriers to care people experience in prison, and especially in solitary confinement, is key to improving their access to care during and after incarceration. Drawing on in-depth qualitative interviews with a random sample of 106 people living in solitary confinement and a convenience sample of 77 people working in solitary confinement in Washington State, we identify two key barriers to care that people in solitary confinement face: cultural barriers (assumptions that incarcerated people do not need or do not deserve care) and structural barriers (physical spaces and policies that make contacting a healthcare provider difficult). While scholarship has documented both the negative health consequences of solitary confinement and correctional healthcare providers’ challenges navigating between the “dual loyalty” of patient care and security missions, especially within solitary confinement, few have documented the specific mechanisms by which people in solitary confinement are repeatedly triaged out of healthcare access. Understanding these barriers to care is critical not only to improving correctional healthcare delivery but also to improving healthcare access for millions of formerly incarcerated people who have likely had negative experiences seeking healthcare in prison, especially if they were in solitary confinement.

## 1. Introduction

In prisons, healthcare is provided at the intersection of individuals’ needs and institutional security restrictions. That is, healthcare assessment and treatment in prison are driven not only by the determination of a person’s mental and/or physical health diagnosis, but they are also shaped and constrained by security protocols. As the following correctional healthcare worker notes, routine intake assessments conducted by medical staff when admitting a person to a solitary confinement unit are used to both identify health needs and determine a person’s suitability for possible uses of force:

No matter where they come from [before arriving in solitary confinement], if there’s no injuries, I still have to do an intake questionnaire and assess whether or not they can be OC’d [be sprayed with oleoresin capsicum vapor] …you know, the spray, or the [cell extraction] shield.

—Kelly [pseudonyms have been assigned to all interview participants to ensure anonymity], Nurse

In this example, and in the many to follow in this article, security needs are given equal priority with, or even treated as superseding, individuals’ healthcare needs, creating barriers to care that impact all parties involved in both the delivery and receipt of care—incarcerated persons, healthcare providers, and correctional staff alike. Understanding how barriers to care shape the experiences and interactions of people who live and work in solitary confinement can provide key theoretical insights into the health impacts of incarceration and help inform the professional ethics of providers.

To understand the relative importance of unpacking barriers to care within carceral facilities, the scope of the problem—how many people experience inadequate carceral healthcare—must be established. The most recent data indicate that there are 2.3 million people incarcerated in U.S. prisons, but this number is only the figurative tip of the carceral iceberg—4.9 million people are formerly incarcerated in the United States, and 113 million have an immediate family member who has been incarcerated [1]. The healthcare implications of this scale of prison system contact are just beginning to be understood. People in prison are at higher risk than the general population for a wide range of infectious diseases (HIV and Hepatitis C), disorders (substance use and psychiatric), and chronic illnesses (obesity, hypertension, and asthma) [2,3,4,5]. Because racial minorities are disproportionately likely to experience incarceration in the United States, the experience of incarceration itself is now considered a negative social determinant of health, especially for Black men [6].

Within this carceral landscape, solitary confinement constitutes the most extreme form of confinement and has severe implications for health. Although the overall number of people in solitary confinement in the United States on any given day has been falling (from an estimated 68,000 in 2016 to an estimated 31,000 in 2020) [7], the number of people who have spent some time in solitary confinement at some point during their incarceration is increasing. As many as one in five people incarcerated across the United States has spent time in solitary confinement in the past year [8]. In our work in Washington State, the proportion of people who had ever spent time in solitary confinement was even higher: one in three people incarcerated in the prison system had spent time in solitary confinement during their incarceration [9]. People who spend even a few weeks in solitary confinement, locked into small, often windowless cells for 22 or more hours a day, can experience adverse mental and physical health outcomes including higher rates of self-harm [10] and suicide [11], a higher cardiovascular health burden [12], and potentially shrinking hippocampi [13]. People in solitary confinement are also disproportionately likely to be Black and Latinx [6,9,14,15,16,17,18]; these groups, then, bear a disproportionate burden of the negative health outcomes associated with time in isolation [19]. A recent study also found that, beyond the immediate severe and detrimental health consequences of isolation, people who had experienced solitary confinement were substantially more likely to die after release from prison—by suicide, homicide, and opioid overdose—than those who had not experienced such conditions of confinement [20].

Incarceration, and especially incarceration in solitary confinement, is a risk factor that healthcare providers need to better understand and address when providing care not only in carceral settings, but also in the general community. While a growing body of literature has documented the adverse and racially disproportionate outcomes associated with incarceration and, especially, solitary confinement, less attention has been paid to how solitary confinement itself impedes the provision of healthcare. Some have described the difficulties of administering (or attempting to administer) mental health care at the window of a solitary cell door [19,21,22,23]. Others have described the ethical challenges confronting healthcare providers forced to choose between patient care and personal or institutional security (“dual loyalty”) [24,25]. However, few have systematically identified the multiple mechanisms thwarting care-seeking and treatment for people in segregation.

Here, we apply the theoretical framework of institutional logics to the context of prison healthcare to understand why incarcerated people do or do not seek out and receive care, and to explain how practitioners and advocates can ultimately improve care for currently and formerly incarcerated populations. Institutional logics reflect the frames of reference that shape rules in an organizational setting as well as the values, beliefs, and practices of organizational actors [26]. Within criminal justice research, institutional logics have been used as a framework to examine: drug court perceptions and operations [27]; how organizational actors make sense of and respond to litigation and federal reform efforts concerning consensual sex and sexual assault in prison (e.g., PREA) [28,29]; and the limits of solitary confinement reform—and penal reform more generally [30]. The specific logics identified vary by study—ranging from organizational priorities such as custody, control, care, rehabilitation, and efficiency, on the one hand, and cultural beliefs such as prisoner-staff mistrust and prisoner degradation on the other. Among these varied logics though, most studies find that the institutional logic prioritizing safety and security undermines reforms attempting to centralize the well-being and rights of the incarcerated. This paper builds upon these analyses by identifying and analyzing the institutional logics governing and constraining the provision of prison healthcare—specifically, access to and administration of healthcare within solitary confinement.

Healthcare reform within correctional settings has been an ongoing battle for the past two decades [31,32], and access to healthcare is one of the most distinct constitutional rights that incarcerated people are granted under the law. Yet, as Rudes et al. have documented [28], carceral logics can prove dismissive of and even hostile to the provision of care in prisons in ways that violate prisoners’ civil rights—whether a right to recourse for sexual violence or a right to care. Legal mandates that prisons provide adequate healthcare are therefore at risk of being subsumed by institutional logics that prioritize the securitization and dehumanization of prison life over the health and well-being of incarcerated persons [21,28]. By leveraging an institutional logics framework, scholars and practitioners can more readily identify the boundaries of prisoners’ rights, as well as the organizational mechanisms (modes of thought, interpretation, and interaction) that drive the adverse health outcomes of incarceration.

In this article, we extend prior work on the consequences of solitary confinement for individual health by examining the impediments that this practice creates for the delivery and receipt of correctional healthcare. Specifically, we pivot from a focus on solitary confinement’s consequences for individual health outcomes to the impediments it creates for the delivery of correctional healthcare, impediments sustained by the intensification of the institutional logic of securitization in solitary confinement. Indeed, systematic analyses of care-seeking and treatment for people in segregation are limited. Studies of solitary confinement also rarely integrate the perspectives of both staff and the incarcerated. Drawing on in-depth qualitative interviews with a random sample of 106 people living in solitary confinement and a convenience sample of 77 people working in solitary confinement in Washington State, we identify two categories of barriers that stymie both the provision and quality of care that people in solitary confinement receive: cultural barriers (assumptions that incarcerated people do not need or do not deserve care) and structural barriers (physical spaces and policies that make contacting a healthcare provider difficult). Ultimately, the physical spaces of solitary confinement, combined with cultural norms prioritizing prison security over the well-being of the incarcerated population, repeatedly triage people *out of*, rather than *into*, care. While this happens outside of solitary confinement, too, the experience of being triaged out of care is exaggerated and amplified in the highly restrictive and charged environment of solitary confinement. Understanding how incarcerated people, especially those in solitary confinement, experience these barriers to care can help improve healthcare provision both in prison and for formerly incarcerated people after prison.

## 2. Materials and Methods

### 2.1. Subjects

In this paper, we draw on a total of 263 in-depth qualitative interviews with a random sample of incarcerated people (106 interviews in year one and 80 follow-up interviews in year two) and a strategic convenience sample of staff (77 interviews in year one) living and working in Washington State’s five Intensive Management Units (IMUs). The University of California, Irvine (UCI) Institutional Review Board, and Washington Department of Corrections (DOC) approved the research study, and careful protocols were followed to randomly select and protect vulnerable research participants. First, Washington prison officials provided the research team with a list of all incarcerated people on maximum custody status (the highest security status) in a given IMU within a few days prior to the research team’s visit to each of the state’s five IMUs. Second, a research team member then randomized the list to determine an order of approaching potential research participants. At each of the five IMUs, our target goal was to interview roughly one-third of maximum custody status prisoners (for a total of roughly 100 participants out of 300 total eligible). Then, to recruit participants, a research team member approached potential participants at cell-front, explained the study, informed potential participants that participation was voluntary, would not involve incentives (administrative or otherwise), that participation refusal would have no adverse effects, and noted whether the prisoner would be interested in participating. Willing participants were escorted one-by-one to a confidential area (monitored visually but not aurally by DOC staff), consented (including reiteration of the voluntariness of participation and explanations that all information shared would be protected and anonymized unless it pertained to “an imminent security-related threat”), and interviewed by one or two members of the research team. (For additional steps taken to protect vulnerable imprisoned research participants and details of the training research team members completed, see Appendix A of Reiter et al., 2020 [33].) In all, 106 incarcerated people participated in interviews; 39 percent of those approached for participation refused, which is comparable to similar studies of incarcerated people [34,35]. In year two, the research team attempted to re-interview all of the year-one participants who were still incarcerated within Washington DOC. In total, we conducted 80 re-interviews. Only 4 participants refused re-interviews; 1 died; 21 were unavailable because of institutional transfers or release to parole. This drop-out rate is low compared to similar studies [36,37]. The random sample of incarcerated people in IMUs we interviewed was not significantly different in terms of demographic variables or criminal history characteristics from the overall population of people in Washington’s IMUs [33].

Unlike incarcerated participants, staff were not randomly selected for interviews during year one. Rather, the research team identified a strategic convenience sample of custody (correctional officers and supervisors) and non-custody (program and healthcare providers) staff. Efforts were made to interview custody staff from all three shifts (morning, afternoon, and night), non-custody staff, and supervisory staff at all five facilities. Staff were informed ahead of time about scheduled interview trips and encouraged by DOC administrative leadership to participate if they felt comfortable. Once on site at each facility, research team members directly approached staff (usually in the afternoon or on the second day of interviews on site after the work of identifying and moving incarcerated participants into interview rooms was underway) to identify willing staff participants. Staff were informed that participation was voluntary and would not involve incentives, administrative or otherwise; that refusal would not affect them adversely; and that all information shared would be protected and anonymized. In all, 77 staff from across all five IMUs and headquarters participated in interviews. Staff interviews at each facility incorporated at least two IMU healthcare providers including nurses, physician assistants, and/or psychology associates. Since staff were strategically sampled, and many staff interviewed worked both in the IMU and in other units within the prison, a refusal rate cannot readily be calculated for the staff interviews; likewise, because we did not have data about the overall characteristics of staff across DOC, we cannot calculate how the demographics of our staff subjects compared to the demographics of the overall staff population. Interviews were conducted in 2017 and 2018. (For more data about overall interview characteristics, see Reiter et al., 2021; Reiter et al., 2020 [33,38])

### 2.2. Data Collection Procedures

Interviews were conducted face-to-face in private rooms and followed a semi-structured interview instrument including 70 (for staff) and 96 (for incarcerated people in year one) questions concerning conditions of daily life, physical and mental health, programming available in the IMUs, and demographics. Interviews lasted between 30 min and three hours and averaged 90 min, were recorded with subjects’ permission, transcribed, stripped of identifying details, and uploaded into Atlas.ti qualitative analysis software (ATLAS.ti Scientific Software Development GmbH: Berlin, Germany) for analysis. Pseudonyms were assigned to participants to preserve anonymity.

### 2.3. Qualitative Analyses

For our qualitative interview data, we used a thematically grounded, open-coding process [39]. To develop a codebook for analyzing these hundreds of hours of interview data, six team members open-coded 24 transcripts (4 each) line-by-line, inductively exploring how participants understood restrictive housing, generating an initial list of over 500 codes. These codes were further refined and categorized, then condensed into 176 codes, organized into ten thematic code groups, including the two we drew on in this article: IMU Relations and Health. After a round of pilot coding, in which each team member completed one initial transcript coding and one recoding, coding discrepancies were reconciled. Team members then coded within code groups of interest, such as “Health”. Coders met bi-weekly for 6 months to resolve discrepancies. Codes were applied across interviews with both incarcerated people and staff. Given this intensive, thematically-grounded process, no statistics were calculated for intercoder agreement.

To understand how our incarcerated sample accesses and receives care, and how staff perceive and provide care to those in the IMU, we analyzed data corresponding to the “health” and “IMU relations” code groups. Specific codes analyzed include: diagnosis, healthcare, treatment, medication, mental health, manipulation, discretion, behavioral interpretation, trust, and custody/care tension. Each code came up hundreds of times across our interviews with both incarcerated people and staff; an analysis of specific quotes relating to access in healthcare generated the findings about cultural and structural barriers to care explored below.

These themes about barriers to care were pervasive in the data, coming up every time access to healthcare was discussed by either incarcerated people or staff. We use representative examples from this rich data to define and explore both the genesis and implications of these barriers, as a starting point for establishing a foundation of understanding the challenges to providing care in solitary confinement, in prison, and beyond. As noted, we applied the same codes to interviews with both incarcerated people and staff and identified extraordinarily similar themes across these two participant groups. In our presentation of findings, we integrate examples from interviews with both incarcerated people and staff to illuminate the consistency in the institutional logics that govern both experiences with and decisions about care in solitary confinement. Indeed, our data reveal that, in spite of the common presumption of conflict and oppositional views between prisoners and staff, their perceptions of the barriers to care are remarkably consistent.

## 3. Results and Discussion

While the problems with providing healthcare in prison are well-documented [28,31,32,33], as are the negative consequences of solitary confinement on health and well-being [10,11,12,13,19,22], less attention has been paid to the specific barriers to providing care in this setting. The intensely controlled nature of solitary confinement units, where people leave their cells only in hand and leg cuffs and are escorted by two or more officers at a time, renders access to care entirely dependent on informal screening by staff. Similar to intake specialists in an emergency room, correctional officers assess the severity and legitimacy of complaints and ailments before deciding whether or not to refer an incarcerated person to the next level of care—be that a nurse, doctor, or mental health specialist. In sum, in solitary confinement units, such as Washington state’s IMUs, correctional officers function as triage paraprofessionals. However, unlike nurses or other medical staff, correctional officers are marginally trained (if at all) to identify and respond to visible signs of physical and psychological distress. Instead, officers are trained to prioritize the safety and security of a facility—goals that are intensified in the high-security, highly restrictive solitary confinement setting. Our interview data reveal how, in restrictive housing units in Washington State, these institutional imperatives impacted not only referral or escalation of care decisions, but also whether and how incarcerated people sought care and received treatment. The following sections illustrate how the tension between institutional logics of security, safety, and, also, efficiency, as opposed to care, shaped other cultural norms and interacted with structural realities in solitary confinement units. Together, these cultural and structural barriers not only limited the provision and quality of care that incarcerated participants received within solitary confinement units in Washington State prisons, but shaped how participants sought care and how care providers responded.

### 3.1. Cultural Barriers to Care

People incarcerated in Washington’s IMUs consistently described challenges with “getting help” in these units, pointing to these challenges as one of the harshest and hardest parts of the experience of isolation. As Jordan, a participant incarcerated in the IMU, explained: “The hardest part [of the IMU] is like…the help you feel like you need, it’s hard to get it. And you don’t know how to do it, what to do, and which way to get it”. Jordan and Gabriel both explained that people in IMUs feel forced into taking extreme steps in order to obtain care. Jordan said: “So that’s why a lot of people try to hurt themselves. They try to kill themselves, to go to the infirmary or to go to another institution where they can get help”. Indeed, Gabriel said he had repeatedly “banged [his] head on the metal shower doors” because “every time I tried to get somebody to get mental health to come talk to me, they wouldn’t do it”. Gabriel went on to explain that it wasn’t until he had a head bleed that he was finally able to access care: “Oh, you’re bleeding. Let’s call mental health now”. Incarcerated respondents framed certain acts of self-harm, such as Gabriel’s, as acts of “resistance”, necessary “just to get results”, i.e., medical attention (Michael, Incarcerated Person). Importantly, Michael explicitly acknowledged the existence of manipulation in some prisoners’ actions, describing self-harm as an instrumental act of resistance calculated to get help.

Implicit in Jordan and Gabriel’s stories is an assumption that prison staff will ignore, dismiss, deny, or even outright disbelieve their urgent health needs. Indeed, if people in prison have already lost some credibility by breaking the law, people in the IMU have lost even more credibility, having either broken prison rules or threatened institutional safety and security, justifying their placement in highly restrictive conditions of confinement. People in the IMU, then, as Gabriel and Jordan described, face disbelief about their claims of physical or mental pains. They repeatedly encounter institutional presumptions that they are manipulative, dangerous, and undeserving. This may be especially true for prisoners who have been placed in the IMU for assaulting staff, those who have previously attempted to harm IMU staff, or who have engaged in past self-harm.

Correctional staff, in fact, describe exactly these perceptions of prisoners as manipulative, characterizing instances of prisoner self-harm as instrumental, and reporting symptoms as being exaggerated. For instance, Matthew, a correctional officer, argued that people housed in solitary confinement units “are really into pushing the line”, regardless of mental health needs or documented illness. For this officer, “eating spoons, sticking pens up [one’s] penis, eating shower shoes, or self-harming” reflected “smart” instrumental behavior (i.e., malingering) rather than psychosis. Indeed, he suggested that individuals in the IMU would purposefully threaten to self-harm during “a shift change … when it’s least convenient for us or other guys that are living there”. These tensions around believability and symptom severity were particularly salient with calls for mental health attention. For Matthew and other staff, there was an assumption that rationality and psychosis did not and could not co-exist. This zero-sum approach to mental health distress (i.e., you are either entirely rational or entirely psychotic) not only foreclosed opportunities for care among those in the IMU, it also prevented staff from envisioning the institutional logic of safety as being inclusive of ongoing care.

Notably, incarcerated participants Jordan and Gabriel admitted, just as officer Matthew asserted, that people in the IMU did indeed engage in actions that were instrumental, harming themselves in order to establish sufficient severity of distress to attract or “earn” medical attention. Even when people in the IMU managed to overcome correctional logics of downplaying and dismissing prisoners’ distress (as exhibited by Matthew’s comments above), the attention they did receive could hardly be called “care”. IMU policy permits correctional officers to dispense a substance akin to pepper spray when a resident is engaging in self-harm and is unresponsive to commands to stop, stand, and/or “cuff up”. People in the IMU largely viewed this tactic as “cruel and unusual punishment” (Spencer, Incarcerated Person). Prison policies authorizing the use of pepper spray during prisoner self-harm incidents not only seem punitive, they also position incarcerated residents as a security threat first, and a person in distress to be cared for second. Officers explicitly characterized use of pepper sprays in these situations as an “emergent” use of force, meant to keep prisoners “safe” from themselves (Daniel, Correctional Officer). The cycle of self-harm met with force spirals when IMU residents such as Michael admitted to self-harming to get attention or care; correctional officers then have evidence for their assumptions, bolstering the institutional logic that self-harmers are malingers. This allows staff to justify their rationale for ignoring subsequent requests for assistance, writing off subsequent acts of self-harm and effectively triaging incarcerated people “out” of care.

Cultural assumptions of resident (un)believability also extend to physical health concerns. People incarcerated in the IMU must send “kites”, or written requests to see medical personnel, and obtain approval before a trip to the infirmary is made. Greg (an IMU resident), described how multiple requests for medical care were often made to no avail; instead, requests were frequently dismissed as attempts to manipulate the system to his advantage. He explained:

I had osteomyelitis in my bone, and it destroyed my joint. So, my finger is not fused. It’s just sitting there. After the bone infection healed up or whatever the deal is, I had a compound fracture in my knuckle, so it doesn’t move at all.

Greg repeatedly requested to have his finger fused straight or, alternatively, amputated; however, administrators refused these requests, repeatedly claiming that nothing could be done as the injury was not life threatening. During the interview, Greg also said he had been told by staff that he received “too much” medical treatment during his incarceration, suggesting that there is a limit to how much healthcare a person can receive in prison.

As with requests for mental healthcare, even healthcare providers in prison echoed the IMU residents’ claims, describing how often medical needs were simply ignored. For instance, in one interview, a nurse explained that she was unable to provide an IMU resident with a catheter because he had a prior history with self-harm, and specifically attempts at hanging himself (Debbie, Nurse). A nurse at another facility noted that residents with histories of making weapons are not permitted to keep a rescue inhaler in their cell. Though she said she could make it down to a person’s cell “in about three minutes” should a request be made, three minutes is also enough time for a person to hyperventilate and fall unconscious (Kelly, Nurse). Though logical for the context, these types of institutional policies prioritize risk minimization and prevent staff from being able to provide adequate care, even for conditions as common as asthma.

In sum, people incarcerated in the IMU described difficulties obtaining basic care for mental and medical health needs. Incarcerated participants reported that their requests for care were often dismissed or misinterpreted, pushing them to escalate their requests in ways they acknowledged were calculated in order to establish the severity of their distress and force a response. Correctional officers and healthcare staff, likewise, described IMU residents as dangerous, manipulative, and undeserving. These cultural logics—a specific subset of dominant institutional logics of security and control—were acknowledged and described by both residents and staff in solitary confinement, and these logics inhibited both residents from obtaining and staff from providing care.

### 3.2. Structural Barriers to Care

The institutional logic of security and the cultural norms described above reinforce existing structural barriers to care in restricted housing units, particularly around daily movement patterns, low staffing levels, and the lack of adequate in-house medical facilities. As a condition of their confinement in segregation, residents’ movements are severely limited (23-h lockdown) and hyper-surveilled. When they do leave their cells, people in solitary confinement are always escorted by two to four correctional officers. Custody staff follow strict protocols in transporting carceral residents to yard, showers, the infirmary, visitation, and back to their cells. IMU residents described how these physical and policy restrictions on movement often inhibited, or even entirely prevented, them from getting basic medical care.

Incarcerated participants described diagnoses with terminal illnesses, including leukemia, heart disease, and cancer. These residents consistently expressed frustration with their inability to receive medical attention for these established terminal diagnoses, pointing to the physical limitations on both getting staff attention and getting to medical care facilities from the IMU. For instance, Emilio, a prisoner diagnosed with leukemia, highlighted the unavailability of care in the IMU as compared to the general prison population:

In here, I feel like I don’t get the right treatment for my illness. And if I go to [general] population then things will be different. But see? That’s why I never challenge anything, because [correctional officers] hear you, but they never do anything about it.

Emilio elaborated, describing how living in an IMU added another level of security and set of limitations to his daily routine. He described how IMU policies contributed, first, to a worsening of his physical condition. Even for healthy prisoners, the conditions of isolation (including poor nutrition, deprivation of goods, and lack of physical movement) may erode health and increase the likelihood of developing new illnesses [19]. For someone like Emilio with a terminal illness, such conditions could be more dangerous, especially without access to regular medical care.

Emilio described how IMU policies, second, impacted his ability to receive appropriate medical care. At the time of the interview in 2017, Emilio had only been able to meet with an off-site oncologist twice in the prior 12 months. Medical practitioners might have created a healthcare plan for Emilio, but prison administrators would have had to approve it, and custody staff would be needed to carry it out. If there are not enough staff to transport a prisoner such as Emilio from the IMU to a medical appointment off-site, then that appointment is compromised and, once again, custody logics prevail. Indeed, Emilio felt strongly that his health care needs were neglected while in solitary.

Other study participants consistently described how staff, correctional and health alike, prioritized keeping costs down. This materialized in a variety of ways, such as delaying or denying services, cheapening treatments, not investing in medical infrastructure, or dismissing some types of care as a “waste of money”. For respondents who required medical attention from a specialized physician outside of the prison, such appointments were routinely delayed as a direct result of restrictive IMU policies. Respondents described how special authorization must be obtained from prison administrators to allow transportation and ensure that enough staff are available to complete the transport without compromising the daily movements of the unit. These regulations inhibited prisoners from accessing consultations or care at the appropriate time. Even within the prison walls, the institutional logic of security extended to the infirmary. IMU residents must remain shackled throughout medical examinations (standard procedure in many isolation units), and a correctional officer must be present in the room during the medical exam. In these cases, doctor-patient confidentiality is disregarded completely. As such, the incarcerated person may not be as forthcoming when reporting symptoms for fear of how they might be perceived, or simply because of privacy concerns.

Medical personnel largely validated the health concerns that prisoners such as Emilio and others voiced. Nash, a registered nurse, described their role in the prison as comparable to working in an urgent care medical facility, where they provide treatment for minor ailments and stabilize patients, but lack the ability to provide any diagnostic testing and have only limited ability to provide ongoing treatment. Nash explained:

[We see] fighting injuries, earaches, common cold type stuff, sore throats, respiratory stuff, urinary complaints...It can be all across the board for what we could see here. And then also, I manage diabetes and hypertension. We have an X-ray tech, but he’s running around all over the place, and is sometimes at other facilities. So we don’t have some of the rapid-type testing for blood disorders available here. If we want it stat, we have to send it down to the hospital, which costs time and money. So we don’t have as much diagnostic ability right here.

The lack of infrastructure and resources in prisons, and even more so in solitary confinement, constrains the medical staff’s ability to provide care to patients. For example, even for someone such as Emilio, who was diagnosed with a serious medical condition that required ongoing treatment, few prisons had in-patient medical facilities adequate to administer such care. As described by Hollie, a nurse:

So, if we have somebody who needs IV antibiotics for a couple of days—we’ll try everything here. We’ll give them [intramuscular] injections. We’ll try to hang onto the guy as long as we can, safely. Or maybe he just needs some IV fluids and we’ll perk him up and get better.

Hollie explained how she does her best to provide a modified version of the recommended course of treatment, but that she lacks the necessary resources. She emphasized being mindful of “everybody’s” safety, but also described how patients may ultimately receive care that only temporarily alleviates their symptoms (fluids to “perk up”), rather than prescribed treatments (IV antibiotics), which may be delayed or never provided.

Mental health care providers described similar structural constraints on their abilities to care for IMU residents. Mental health disorder rates are higher in prison than in the general population, and they are higher still in solitary confinement units [33], leaving mental health staff spread thin. With high caseloads and frequent emergency incidents, care is administered as needed under the constraints of rigid security protocols. For example, one mental health director interviewed had 80 patients on their current caseload. As a result, they were only able to meet with individuals once every few weeks; care was often further delayed by the necessary prioritization of emergency incidents, namely incarcerated patients who declare a self-harm emergency. This sporadic mental health care does not allow for meaningful ongoing treatment, and instead is a superficial intervention aimed at helping the individual “get through it”. Further, as with physical health, the conditions of confinement contribute to new or worsening mental health for people incarcerated in solitary confinement, but healthcare staff are unable to address this environmental source of harm. Marie, a psychologist, described the futility she felt in trying to provide mental health care to patients in segregation:

From an institutional perspective, it’s very hard for a mental health staff to talk a patient into doing something different when the environment is the same. It’s like doing therapy for a child but not doing therapy with the parents.

Further, movement to a unit’s mental health office must be timed so as not to interfere with daily operations. Unless there is a life-threatening situation (which, as explained above, requires discretion and legitimization from custody staff), calls for healthcare often come second to these daily activities. As Dave, a psychology associate, explained:

Dave: When emergency procedures are going on, pretty much the rest of the activity shuts down: medical, mental health, all that stuff.

Interviewer: How are you supposed to do your tier checks when there’s OC [oleoresin capsicum] being sprayed.

Dave: Correct. So, that interferes. And I think that, and this has been a long-standing issue up there, that this IMU is staffed pretty much for movements like showers, meals and yard outs and stuff like that. And so on top of that, there’s a little bit of mental health, there’s a little bit of psychiatry, and there’s a little bit of sick call. You know, but there’s not always just people available to transport guys for every possible need. Like, if I get a kite from a guy who says, “Geez, I really need to talk to you”, it depends on if I can get him down here or not. And I’m not in control of that. So, we have disciplinary segregation going on, hearings going on, and you know something happened in another part of the IMU that’s a problem, then I don’t have escort staff.

Dave describes how his ability to respond to requests for treatment is constrained by the other activities occurring in solitary confinement units, including the availability of security staff to assist with prisoner transport to care. Requests for care are constructed as disruptive to daily life in restrictive housing, rather than a pressing aspect of it. These other activities, and the resources and staffing required to conduct them, take precedent over prisoners’ healthcare needs and concerns, even when cultural obstructions (e.g., suspicion of malingering) are not at play.

Because of the institutional hierarchies within prison, mental health staff have no authority to impact security operations or correctional staff behavior, even when the environment itself is a risk to their patient. This reality leaves little opportunity for mental health staff to provide effective mental health care. They instead are left with limited, short-term solutions. For example, some mental health workers described resorting to advocating for patient transfers to facilities with more specialized mental health units in an attempt to avoid counterproductive interference from custody staff, via agitation and escalation. Staff psychologist Marie illustrates this point:

There was one situation where an inmate was taken out of his cell and they used OC spray. He covered his cell door and was acting out and created weapons. He had agreed to come out, but then at last minute, stuck his hand through the cuff port, interpreted as use of force, so they sprayed him with OC. They did what they have to do. They automatically decontaminated. They put him in another cell. He was starting to get agitated because he didn’t have a smock. And so, he just got increasingly agitated, and they used that as justification to not give a smock. So, I did talk to the captain about this, and the superintendent. Like, they might be able to justify that per policy, but is it the right thing to do? So, when I ended up getting that person transferred, it was because they had decompensated, but I also knew that they were not going to be able to have any stability in our IMU.

In this example, the mental health staff member transferred their patient out of the IMU, not to necessarily receive better treatment, but to remove them from the immediate damaging environment. This does not address any of the mental health concerns for which the patient was initially being treated, but instead acts as a band-aid fix for situations the mental health staff member cannot control. Worse, transfers between facilities can keep IMU residents in the restrictive conditions of solitary confinement even longer. People incarcerated in the IMU usually must complete a combination of programs as a condition of their release back to general population, but transfers can interrupt or re-start this program participation, especially if their new facility does not have space in the programs needed to exit solitary.

Institutional logics of security and control are reflected in how physical space and resources are utilized in the IMU (see also Augustine et al., 2021 [21]). The administration of policy, staffing allocations, and the everyday patterns and routines of the IMU place significant strains and limitations on the provision of care. In turn, care-seeking is constructed as disruptive and only necessary during moments of emergency. Medical and mental health crises are something to manage and contain, whereas therapeutic and quality-of-life treatments are subjected to delay and denial. Both health staff and incarcerated participants share strikingly similar perspectives in this regard. Whether one is considered a patient or provider, structural barriers to care reinforce the predominance and prioritization of carceral logics.

### 3.3. Limitations of the Study

Our study represents one of the larger (in terms of number of interviews conducted: 263) and more rigorous (in terms of randomized selection of incarcerated participants) studies of solitary confinement in the literature, but it took place over just two years in one state prison. Washington state has a mid-sized (39th highest rate of incarceration in the United States) corrections department with a long history of inviting and sustaining researcher-practitioner collaborations [9,33], as well as working to reform solitary confinement (for an overview of reform efforts, see the Washington Department of Corrections Restrictive Housing reform overview [40]). The experiences of people living and working in Washington state IMUs, then, may not be representative of the experiences of incarcerated people and staff in other solitary confinement units in other state prison systems. However, our purpose in this piece is not to claim a universality of experience but to begin to identify the kinds of barriers to care people face in solitary confinement, as a starting point for developing mechanisms to redress these barriers during and after incarceration.

## 4. Conclusions

Research has overwhelmingly demonstrated that incarceration is associated with and exacerbates negative health outcomes, and that providing healthcare in correctional settings is challenging at best [25,41]. This is particularly true for solitary confinement, an experience which has been linked to both physical and mental comorbidities and that presents particularly acute healthcare provision challenges. In this article, we have documented and specified carceral dynamics and logics that undercut the patient-provider relationship in solitary confinement. As discussed, people in isolation face various barriers to accessing constitutionally mandated healthcare. Correctional officer attitudes, unclear bureaucratic policies, and carceral logics that prioritize security and assume a general distrust of prisoners together thwart the adequate provision of mental and medical health services and effectively deny incarcerated people adequate care.

To understand the complete picture of healthcare in the IMU, it is necessary to put the interviews with incarcerated people, security staff, and clinical staff into conversation. When analyzed separately, each source provides reports of cultural and structural barriers to healthcare access and treatment for incarcerated people. However, when analyzed together, the underlying institutional logics that create or reinforce these barriers become legible. Further, each group’s experiences and perspectives confirm those of the other group. Prior research has shown incarcerated people to be accurate in their self-reports [42,43]—a finding reinforced by the consistency of reports across resident and staff interviews in this study.

By examining cultural and structural barriers to care within restrictive housing, our study advances the theoretical application of institutional logics to carceral settings. Not only have we shown how institutional attitudes and beliefs are perpetuated across a range of actors (i.e., correctional officers, health staff, and incarcerated people), but that such frames and dispositions are reinforced by the material conditions and patterns that structure the interactions between staff and incarcerated people. Indeed, our study reaffirms that certain carceral logics take precedent over other institutional goals, such as care, rehabilitation, or prisoners’ rights. Moreover, we expand upon theories of institutional logics by showing how they are reified even by those who do not benefit from their operative dominance. Whether it be health staff who must prioritize patients in mental health and medical crises or an incarcerated person who purposely escalates their situation (e.g., engaging in self-harm) so that they are treated as an emergency case, both instances reproduce carceral logics of security and control. Health staff are unable to fully engage professional practice and ethics, while incarcerated people either discourage themselves from care-seeking or, should they choose to pursue care, risk perpetuating stereotypes of malingering and distrust.

Identifying and analyzing the institutional barriers to delivering a robust course of care to people who live in solitary confinement makes a critical contribution to healthcare and equity discussions. Scholars, practitioners, and policymakers are increasingly acknowledging the threat that incarceration poses to public health in the wake of the COVID-19 pandemic. Indeed, some of the worst outbreaks have occurred across jails and prisons, prompting legal action on the part of incarcerated people and their advocates. These efforts have extended prisoners’ civil rights claims to adequate healthcare, centering the physical and psychological harms posed by the “prison within a prison” that is solitary confinement. While public health discourse offers a critical reframing of the harms of incarceration, our study highlights how carceral logics might frustrate and disrupt these developments from securing widescale institutional change.

Our work also extends critical insights into the ethical problems posed by carceral care. Specifically, we elaborate upon the issue of dual loyalty whereby the medical expertise and duties of healthcare providers become overridden by the interests of the prison [23]. As an “omnipresent force” [25], dual loyalty arises when health staff clear prisoners for placement in solitary confinement or when they perceive patients within such settings as goal driven. Still, dual loyalty is primarily framed by the active participation of health care staff within carceral institutions, which leaves their capacity for ethical intervention unexamined [44]. Here, our findings help to consider the ways in which health staff acquiesce to the institutional logics of solitary confinement. Whereas reformers might want health staff to assume a more operative role, an increased level of authority may capitulate to the institutional logics of punitive control. In addition, while the institutionalized passivity of health staff is concerning from a clinical perspective, their mere presence might nonetheless fulfill ethical, albeit compromised, roles, such as bearing witness to the harms of isolation.

In his 2020 ethnography, Siem describes how patients seeking emergency care are treated as burdens “shuffled” between police officers, ambulance personnel, and emergency room medical staff [45]. Our work reveals how, instead of burdens to be “shuffled” between responsible parties, people in solitary confinement are treated as burdens to be triaged out of care entirely, their medical concerns morphing into mechanisms through which staff can reassert the priority of security and, at times, reinforce existing cultural assumptions about prisoners (e.g., manipulative and untrustworthy). These findings help to elaborate the institutional tensions between being a prisoner and patient in solitary confinement. While institutional medicine is generally defined by its capacity to sort and categorize bodies and ailments, solitary confinement, where prisoners are labeled and controlled according to notions of security and risk, complicates this institutional sorting. Carceral categories (i.e., prisoner, threat, and malingerer) prove incompatible and unadaptable to those of care (i.e., patient, illness, and emergency). In turn, prisoners are left with few, if any, viable options for securing care. In the absence of care, they may give up on accessing treatment altogether, wait until their health concerns have worsened, or attempt to exacerbate the situation themselves.

Each outcome is critical to understand for advocates seeking to provide adequate treatment later in the person’s incarceration or after their release from prison. People who have been in solitary confinement may have learned to strategically frame or heighten their symptoms in an attempt to be triaged into treatment. Or, alternatively, they may have learned that seeking healthcare leads to punishment in solitary confinement and therefore may be reluctant to honestly disclose their needs. Incarceration, and especially solitary confinement, distorts the health and needs of those who experience it. Familiarity with these kinds of patterns of (mis)treatment will improve the provision of care for incarcerated and formerly incarcerated individuals.

## Data Availability

Data cannot be shared publicly due to highly sensitive and ethical considerations. Data pertains to prisoners’ experiences and other identifying information. None of the data was approved to be shared outside of the research team. The authors of this paper are happy to provide supplemental material containing additional quotations if deemed necessary by *Healthcare*.
